# Antibacterial Effects of Synthetic Plantaricins Against *Staphylococcus aureus*

**DOI:** 10.3390/antibiotics14030311

**Published:** 2025-03-17

**Authors:** Seung-Eun Oh, Sojeong Heo, Gawon Lee, Jina Kim, Mi-Sun Kwak, Do-Won Jeong

**Affiliations:** 1 Department of Food and Nutrition, Dongduk Women’s University, Seoul 02748, Republic of Korea; ose0110@gmail.com (S.-E.O.); hsjeong325v@gmail.com (S.H.); 20234914@dongduk.ac.kr (G.L.); 2 Insight View Tech, Hwasung 18469, Republic of Korea; jina.kim@insightviewtech.com; 3 Kookmin Bio Corporation, Seoul 02826, Republic of Korea; mskwak@kmbio.co.kr

**Keywords:** plantaricin, *Staphylococcus aureus* ATCC 12692, antibacterial activity, growth inhibition, cell lysis

## Abstract

**Background/Objectives:** Plantaricins without a signal sequence were synthesized based on bacteriocins, plantaricins A, E, F, J, and K, of *Lactiplantibacillus plantarum* KM2. The antibacterial activities of four combinations of synthetic plantaricins—spPlnA, E&F, E&J, and J&K—were identified against *Staphylococcus aureus* ATCC 12692. And in this experiment, we aimed to identify the antimicrobial mechanism of the synthesized plantaricin sample against *S. aureus*. **Methods/Results:** The minimal inhibitory concentrations for each combination were 1.4 μg/mL, 1.8 μg/mL, 1.6 μg/mL, and 1.6 μg/mL, respectively. Raman spectra changed after treating *S. aureus* ATCC 12692 with synthetic plantaricins. Furthermore, transmission electron microscopy results revealed that the four synthetic plantaricin combinations could induce the cell lysis of *S. aureus* ATCC 12692. Finally, the four synthetic plantaricin combinations maintained their antibacterial effect at temperatures below 40 °C, and at pH levels of pH = (4–7). Except for spPlnJ&K, they are stable against the action of α–amylase and lysozyme. Overall, these results indicate that, excepting spPlnJ&K, the three synthetic plantaricin combinations exhibit similar antibacterial activity. **Conclusions:** Through this study, we confirmed that synthetic plantaricin exhibited antimicrobial activity against *S. aureus*, demonstrating its potential as a direct antimicrobial agent. However, since the antimicrobial activity decreased due to protease, it was confirmed that its use is limited in environments where protease is present.

## 1. Introduction

*Staphylococcus aureus* has a long-standing history as a notorious food poisoning bacterium [1]. First identified in the late 19th century by Sir Alexander Ogston, it has since been recognized as a significant cause of foodborne illness worldwide [2]. *Staphylococcus aureus* contamination typically occurs through human handling or through contact with contaminated surfaces, equipment, or raw materials during food processing [3,4]. Once ingested, *S. aureus* can produce heat-stable toxins, such as enterotoxins, which are responsible for the rapid onset of symptoms that include nausea, vomiting, abdominal cramps, and diarrhea [1,5]. Despite its ubiquity, *S. aureus* poses a particular challenge in food safety, due to its ability to survive and thrive in a wide range of environmental conditions, including high salt concentrations and acidic pH levels [6,7]. Moreover, its ability to form biofilms on food contact surfaces further complicates control and eradication efforts in food processing facilities [8]. Given its resilient nature and the severity of its associated illness, effective preventive measures, such as stringent hygiene practices and proper temperature control, are essential to mitigate the risks posed by *S. aureus* in the food supply chain [9].

In clinical settings, *S. aureus* presents significant risks as a pathogen, contributing to a wide array of infections that range from mild skin conditions to life-threatening diseases [10,11]. Its ability to produce an arsenal of virulence factors, including toxins, enzymes, and adhesins, enables it to evade host immune responses and cause diverse infections [12,13,14]. Among the most concerning manifestations are skin and soft tissue infections, such as cellulitis, impetigo, and abscesses, which, if left untreated, can progress rapidly [15,16,17]. Moreover, *S. aureus* is a major cause of healthcare-associated infections, including surgical site infections, bloodstream infections, pneumonia, and device-related infections, leading to significant morbidity and mortality. Of particular concern is the emergence and spread of antibiotic-resistant strains, such as methicillin-resistant *S. aureus* (MRSA), which severely restricts treatment options and complicates infection management [18]. Therefore, proactive measures to prevent S. aureus contamination and infection are crucial.

Bacteriocins represent a promising tool to prevent or control *S. aureus* infections, due to their potent antimicrobial activity and potential for the targeted killing of specific bacterial strains [19,20]. Most importantly, unlike antibiotics, bacteriocin treatment does not generate bacteriocin-resistant strains in the target microorganisms [21]. These ribosomally synthesized peptides or proteins are produced by various bacteria, including certain strains of lactic acid bacteria, and exhibit bactericidal or bacteriostatic effects against a wide range of pathogens that include *S. aureus* [19,20,22]. Bacteriocins can selectively target pathogenic bacteria while sparing beneficial microflora, making them attractive candidates for use in probiotics or as food preservatives [23,24,25,26]. Moreover, their diverse mechanisms of action—including pore formation, DNA or RNA degradation, and interference with cell wall synthesis—make them effective against antibiotic-resistant strains such as MRSA [27,28]. Harnessing the potential of bacteriocins in various applications, including food safety, clinical therapeutics, and bio-preservation, holds promise in mitigating the risks associated with *S. aureus* infections and combating the spread of antimicrobial resistance [24,29].

In our previous study, *Lactiplantibacillus plantarum* KM2 was isolated from aged Korean beef and demonstrated antibacterial effects against seven foodborne pathogens or spoilage bacteria [30]. Genome analysis revealed the strain-specific presence of the entire plantaricin operon in *L. plantarum* KM2 [30,31]. Furthermore, both the culture supernatant and synthetic plantaricin of the *L. plantarum* KM2 exhibited antibacterial activity against *S. aureus* [30,31]. However, the studies that have explored the antimicrobial mechanisms of plantaricin against *S. aureus* have been limited. Therefore, this study aimed to verify the antimicrobial activity of synthetic antimicrobial substances, synthesized based on the bacteriocin gene present in *L. plantarum* KM2, against *S. aureus* strains. Additionally, we sought to elucidate the antimicrobial mechanisms of these synthesized antimicrobial substances against *S. aureus*.

## 2. Results

### 2.1. Antimicrobial Activities of Synthetic Plantaricins Against S. aureus ATCC 12692

In a previous study, the antibacterial activity against *S. aureus* of five synthetic plantaricins, individually or in combination, was confirmed using the disk diffusion method with various combinations [31]. Among the combinations tested, six combinations (spPlnA, E&F, E&J, J&K, A&E, and A&K) showed activity against *S. aureus*, while seven (spPlnJ, F&J, A&F, A&J, A&E&J, A&F&J, and A&F&K) exhibited weak activity. Although two plantaricin combinations, A&E and A&K, showed antibacterial activity against *S. aureus*, their activity was not higher than that of spPlnA alone, and they required the addition of spPlnE and spPlnK to spPlnA, so they were excluded from the experiment. The combinations spPlnE&F, E&J, and J&K demonstrated higher activity, compared to when spPlnE, F, J, and K were tested individually; thus, spPlnE&F, E&J, and J&K were used in the experiment. To conduct the experiment on synthetic plantaricins against *S. aureus* and determine the minimal inhibitory concentration (MIC) values, MIC analysis was performed for the four samples (spPlnA, E&F, E&J, and J&K), and their MIC values were determined to be 1.4 μg/mL, 1.8 μg/mL, 1.6 μg/mL, and 1.6 μg/mL, respectively. The MIC value of nisin, used as the positive control, was confirmed to be 1.7 μg/mL. These results indicated that the synthetic plantaricins exhibited antimicrobial activity against *S. aureus* at a concentration similar to that of nisin.

### 2.2. Antibacterial Activity Effect of Synthetic Plantaricins on S. aureus ATCC 12692 Using Ra Man Spectroscopy

**Figure 1:**
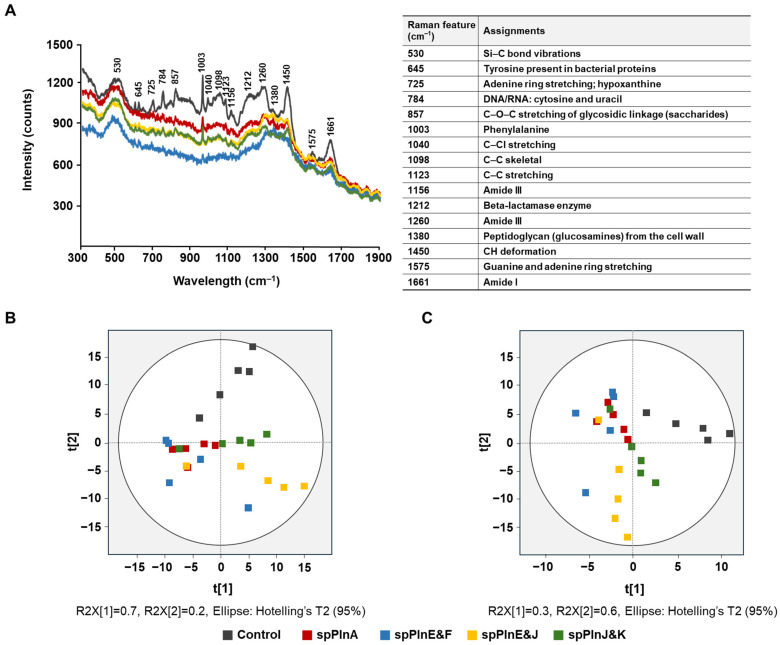
Raman spectra and multivariate analysis of *S. aureus* treated with synthetic plantaricins. Raman spectra (**A**), principal component analysis (**B**), and partial least-squares discriminant analysis (**C**) of *S. aureus* ATCC 12692 treated with synthetic plantaricins. The peak range is from 300 to 1900 cm^−1^. The resulting values of the corresponding peak range were also applied to PCA and PLS−DA [35,36,37,38,39,40,41,42,43].

Raman spectroscopy is a powerful, non-destructive tool for profiling cellular metabolism through spectral analysis [32,33]. It provides detailed molecular insights without requiring extensive sample preparation, enabling the detection of subtle biochemical changes within cells. The Raman spectrum captures molecular vibrations in bacteria, allowing for the prediction of their overall molecular composition. For example, Raman spectroscopy has been utilized to monitor bacterial metabolic product patterns during recent antimicrobial substance treatments, enabling the confirmation of whether the action is antimicrobial susceptibility or antimicrobial activity [34]. In this study, we aimed to demonstrate the effectiveness of Raman spectral analysis in confirming the antimicrobial activity of synthetic plantaricins. Specifically, the synthetic plantaricins spPlnA, E&F, E&J, and J&K were used to treat *S. aureus* ATCC 12692 at concentrations corresponding to half of their MIC values (0.7 μg/mL spPlnA, 0.6 μg/mL spPlnE&F, 0.8 μg/mL spPlnE&J, and 0.8 μg/mL spPlnJ&K). For reproducibility, five independent specimens were obtained and analyzed for each sample, and the spectrum of Raman spectroscopy was derived using their average values (Figure 1A).

The treatment of *S. aureus* ATCC 12692 with plantaricin resulted in a decrease in peak intensities across the entire Raman spectrum compared to *S. aureus* ATCC 12692 that was not treated with plantaricin (Figure 1A). This result indicates the clear impact of synthetic plantaricin on *S. aureus* ATCC 12692. In particular, peaks corresponding to 530, 725, 784, 857, 1003, 1260, 1450, and 1661 cm^−1^ were significantly decreased (Figure 1A) [35,36,37,38,39]. Each band represents Si-C bond vibrations, adenine ring stretching, hypoxanthine, DNA/RNA, cytosine and uracil, C–O–C stretching of glycosidic linkage, phenylalanine, amide III, CH deformation, and amide I, respectively. Additionally, peaks at 645, 1040, 1098, 1123, 1156, 1212, 1380, and 1575 cm^−1^, corresponding to tyrosine present in bacterial proteins, C-CI stretching, C-C skeletal, C-C stretching, amide III, Beta-lactamase enzyme, peptidoglycan (glucosamines) from the cell wall, and guanine and adenine ring stretching, respectively, were also observed to decrease [37,38,40,41,42,43]. These changes imply alterations in the carbon and nitrogen components constituting the cell wall. Such modifications to the spectral bands suggest that the antimicrobial agents affect the bacterial cell wall integrity and composition, potentially disrupting essential cellular processes. The observed variations in Raman peaks provide insights into the molecular interactions and structural changes induced by the treatment, highlighting the effectiveness of synthetic plantaricins in targeting and compromising bacterial cell wall stability.

To enhance the comprehensiveness of our study, we also considered the integration of advanced chemometric techniques into Raman spectroscopy data. Techniques such as PCA and PLS−DA could further elucidate the distinct metabolic changes induced by different plantaricins, thereby providing a deeper understanding of their mechanisms of action. According to Hotelling T2 analysis, no sample was outside the 95% confidence ellipse (Figure 1B,C). To better illustrate the differences in antimicrobial activity among the synthetic plantaricin combinations used for treatment, PCA was applied to the full Raman spectral range (400–1800 cm^−1^). PCA reduces the dimensionality of multivariate data by transforming it into a smaller set of uncorrelated variables, known as principal components (PCs), while retaining most of the variation within the dataset. The untreated control samples, which received no antimicrobial treatment, were positioned in the first and second quadrants, whereas samples treated with antimicrobial substances were distributed in the third and fourth quadrants (Figure 1B). Notably, spPlnE&J exhibited distinct patterns that set them apart from the control group. These findings suggest that synthetic plantaricins have antibacterial effects.

PLS−DA is considered more reliable than PCA for classification purposes [44], particularly for handling complex datasets with multicollinearity and enhancing the interpretability of discrimination between treatment groups. To identify the spectral regions responsible for differentiation, loading plots were generated, highlighting key Raman shifts associated with nucleic acids, proteins, and bacterial cell wall components. PLS-DA further improved classification accuracy, revealing a clear separation between treatment groups and confirming the effectiveness of plantaricins in altering bacterial metabolic profiles (Figure 1C). Samples treated with synthetic plantaricins—spPlnA, E&F, E&J, and J&K—were all located in quadrants 2, 3, and 4, whereas the control remained in quadrant 1, showing a more distinct antibacterial activity (Figure 1C). This separation reinforces the antimicrobial potential of synthetic plantaricins. The statistical robustness of the model was validated using Hotelling T^2^ analysis, confirming that all samples were within the 95% confidence interval, indicating no significant outliers.

### 2.3. Synthetic Plantaricins Cause the Cell Lysis of S. aureus ATCC 12692

**Figure 2:**
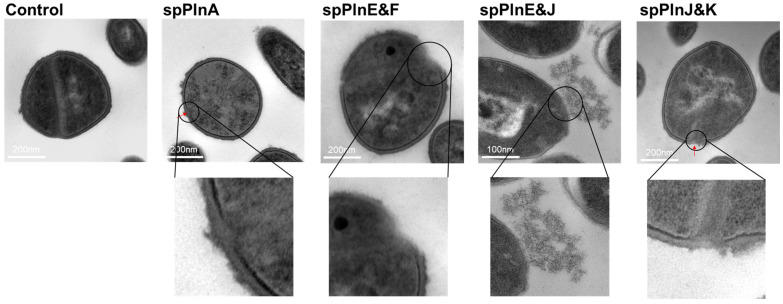
Transmission electron microscopy (TEM) for *Staphylococcus aureus* ATCC 12692 treated with synthetic plantaricins. Bacterial ultrastructure was examined using JEM−1010 transmission electron microscopy (JEOL, Tokyo, Japan). These bacteria were severely affected, particularly by synthetic plantaricins. Debris from the decomposition of the cell wall is marked by red arrows.

Based on Raman spectroscopy results suggesting changes in the cell wall of the indicator strain *S. aureus* ATCC 12692, we aimed to further investigate these effects using transmission electron microscopy (TEM). By correlating Raman spectral changes with antimicrobial activity, we visualized the structural damage in *S. aureus* ATCC 12692 following treatment with four synthetic plantaricin samples (spPlnA, E&F, E&J, and J&K) using TEM. The TEM images revealed the presence of lysis fragments around the *S. aureus* ATCC 12692 cell wall after synthetic plantaricin treatment (Figure 2). Notably, treatment with spPlnA and spPlnJ&K resulted in distinct and clearly visible fragments (Figure 2). These findings suggest that synthetic plantaricins contribute to cell wall damage in *S. aureus* ATCC 12692. These observations are consistent with the Raman spectroscopy findings, confirming the efficacy of synthetic plantaricins in compromising bacterial cell wall integrity and providing a mechanistic understanding of their antimicrobial action.

### 2.4. Effects of Heat, pH, and Enzymes on Antibacterial Activity

The antibacterial activity of the four synthetic plantaricin combinations against *S. aureus* was confirmed to be due to cell wall lysis. The study also aimed to verify the persistence of the antibacterial activity of these combinations against *S. aureus*. It was confirmed that all combinations maintained their activity at temperatures below 40 °C (Table 1). The four plantaricin combinations retained all their activity within the pH range of pH = (4 to 7) (Table 1). Since this pH range is typical for foods, these bacteriocin combinations are expected to have high industrial applicability.

Since plantaricins are peptides, degradation by proteases was anticipated, and as expected, when treated with protease K, their activity was lost. Moreover, when treated with α-amylase and lysozyme, the antimicrobial activity of synthetic plantaricin tended to decrease compared to its activity at pH 7 (Table 1). In particular, the antimicrobial activity of spPlnJ&K significantly decreased when treated with lysozyme. Additionally, the significant differences in antimicrobial activity among the lysozyme-treated samples suggest that the compound is unstable in the presence of lysozyme. On the other hand, while the antimicrobial activity of the α-amylase-treated samples was lower than that at pH 7, there were no significant differences in activity among the samples, indicating that the antimicrobial activity remained stable against α-amylase (Table 1). To determine whether there were any changes in antimicrobial activity during the enzyme inactivation process, an equal amount of synthetic plantaricin, without enzyme treatment, was heated at 100 °C for 5 min, and its antimicrobial activity was then tested. The results show that the antimicrobial activity was not significantly affected, confirming that synthetic plantaricin retains its function as an antimicrobial agent even after being heated at 100 °C for 5 min (Table 1). These results indicate that under common pH and temperature conditions, synthetic plantaricins maintain antibacterial activity and are stable against enzymes other than proteases. If these plantaricins are used in non-sterilized environments, such as food fermentation, they could serve as natural preservatives, suggesting their potential for industrial application. However, since the antimicrobial effect of plantaricin was reduced in the presence of protease, it is important to consider the limitation posed by its antimicrobial activity possibly decreasing in environments where protease is present.

## 3. Discussion

While antibiotics have been traditionally used to prevent or control *S. aureus* infections, their widespread and indiscriminate use has led to several shortcomings. One major issue is the emergence and spread of antibiotic-resistant strains, including MRSA, which, in clinical settings, pose a significant challenge [45]. Additionally, the overuse of antibiotics can disrupt the natural microbiota, leading to dysbiosis and promoting the colonization and proliferation of opportunistic pathogens like *S. aureus* [46]. Furthermore, this can lead to allergic reactions, gastrointestinal disturbances, and the development of secondary infections. Moreover, *S. aureus* has shown a remarkable ability to acquire resistance mechanisms through horizontal gene transfer, further complicating treatment strategies [47]. These shortcomings underscore the urgent need for alternative approaches, such as the development of novel antimicrobial agents or the utilization of non-antibiotic-based interventions, to address the challenges associated with *S. aureus* infections.

Bacteriocins offer several advantages over antibiotics in combating bacterial infections [48,49]. Firstly, bacteriocins exhibit a narrower spectrum of activity, targeting specific bacterial species or strains while sparing beneficial bacteria, thus minimizing disruption to the natural microbiota and reducing the risk of dysbiosis [50]. Additionally, bacteriocins often demonstrate potent antimicrobial activity, even against antibiotic-resistant pathogens, including multidrug-resistant strains, offering a promising alternative in the face of increasing antibiotic resistance [51]. Furthermore, compared to conventional antibiotics, bacteriocins are typically less prone to inducing resistance, as they employ diverse mechanisms of action, including pore formation and enzymatic degradation, making it challenging for bacteria to develop resistance mechanisms [52]. Moreover, bacteriocins can be produced by probiotic bacteria, further enhancing their potential as natural, safe, and sustainable antimicrobial agents for therapeutic and food preservation purposes [53,54].

Several studies have reported the antibacterial activity of plantaricin in controlling the growth of *S. aureus*. Among them, the *L. plantarum* LD1 strain demonstrated antibacterial activity against *S. aureus* using culture supernatant, and the effect was assumed to be due to plantaricin [55]. Similarly, plantaricin NC8 αβ has also been reported to exhibit antibacterial activity against *S. aureus* [56,57]. These findings suggest that plantaricin effectively inhibits *S. aureus* growth, and our results further confirm that synthetic plantaricin also possesses antibacterial properties. Based on these results, further studies are suggested to determine whether *S. aureus* infections can be reduced from a clinical perspective. Additionally, although there have been no reports of increased resistance to bacteriocins, experimental validation is also recommended.

The bacteriocins used in this experiment were synthetic peptides; however, their sequences were derived from the plantaricin sequence of *L. plantarum* KM2, which exhibited effective antimicrobial activity against *S. aureus*. Among them, spPlnE&J demonstrated the strongest antibacterial activity (Figure 1 and Figure 2). While further experiments are needed to determine whether resistance to these bacteriocins may develop, no reports of bacteriocin resistance have been documented so far. Therefore, we proposed that this bacteriocin could be an effective agent for controlling *S. aureus*. In addition to its direct use as an antimicrobial agent, *L. plantarum* KM2, which produces this plantaricin, could be utilized as a fermentation starter culture. By incorporating it into food production, it may help suppress the growth of *S. aureus*, contributing to the development of safer fermented foods. However, since the antibacterial substance used in this study is based on a synthetic antimicrobial compound derived from the genome of *L. plantarum* KM2, it is necessary to confirm the actual presence of plantaricin isolated from KM2 in future studies and to evaluate its production efficiency. Additionally, when using the KM2 strain as a starter culture, it is important to identify other antimicrobial metabolites that may contribute to antibacterial activity beyond plantaricin and to compare their interactions.

## 4. Materials and Methods

### 4.1. Synthetic Plantaricins

In this study, synthetic plantaricins, spPlnA, E, F, J, and K, without signal peptides as previously described [31], were utilized. Briefly, each plantaricin was synthesized using Symphony X (Protein Technologies, Inc., Tucson, AZ, USA) and obtained as a powder, with approximately 30 mg per sample. Mass identity verification was performed using Axima Assistance MALDI–Time of Flight Mass Spectrometry (Shimadzu, Tokyo, Japan). To minimize error differences between samples, all peptide stock solutions were prepared with ddH_2_O; 0.3 mg/mL spPlnA, 0.4 mg/mL spPlnE&F, 0.3 mg/mL spPlnE&J, and 0.3 mg/mL spPlnJ&K. When combining two plantaricins, equal amounts were mixed, and the molecular weights of each were calculated. The average molecular weight was then used to determine the stock concentration.

### 4.2. Bacterial Minimum Inhibitory Concentration

The MIC against *S. aureus* ATCC 12692 was determined by the broth microdilution method. Synthetic plantaricins—spPlnA, spPlnE&F, spPlnE&J, and spPlnJ&K—were prepared by twofold serial dilution in deionized water, and 20 µL was added to each well of a 96-microwell plate to achieve final plantain concentrations ranging from 0.3 to 3.7 μg/mL. *S. aureus* ATCC 12692 were sub-cultured in tryptic soy broth (TSB; Becton, Dickinson and Co., Franklin Lakes, NJ, USA) and matched to a 0.5 McFarland turbidity standard (BioMérieux, Marcy-l’Étoile, France). Each suspension was diluted 1:100 with fresh TSB, and 180 µL was added to each well of a 96-microwell plate. The MIC of synthetic plantaricin was recorded as the lowest concentration at which no *S. aureus* ATCC 12692 growth was observed after incubation at 37 °C for 24 h, and the MIC results were confirmed by at least three independent tests. Nisin (Nisin from *Lactococcus lactis*; N5764, Sigma-Aldrich, Saint Louis, MO, USA) was used as the positive control, and it was dissolved and diluted using Dimethyl sulfoxide (D8418, Sigma-Aldrich). The negative control used was an equal amount of ddH_2_O. All experiments were conducted in triplicate, using independent cultures.

### 4.3. Raman Spectrum

In this study, four synthetic plantaricin samples, spPlnA, spPlnE&F, spPlnE&J, and spPlnJ&K, without signal peptides as previously described [31], underwent Raman spectroscopy. Briefly, *S. aureus* ATCC 12692 was treated overnight with 0.7 μg/mL spPlnA, 0.6 μg/mL spPlnE&F, 0.8 μg/mL spPlnE&J, and 0.8 μg/mL spPlnJ&K—equivalent to half of their MIC values—followed by freeze-drying. The negative control used was the *S. aureus* culture without treatment with synthetic plantaricin. The resulting samples were analyzed by Raman spectroscopy (RAMANtouch; Nanophoton Co., Osaka, Japan) using a single-mode diode laser at 532 nm. A 50× objective lens (numerical aperture 0.5; working distance 500 µm; Nikon, Tokyo, Japan) was used to focus the laser beam. All Raman experiments were performed with a laser power of 2.9 mW and an exposure time of 10 s. To ensure reproducibility, spectral data were collected from five biological replicates and preprocessed using multiple scatter correction and standard normal variate normalization. Finally, the processed data were analyzed using SIMCA-P 17.0 (Umetrics, Umeå, Sweden) to perform principal component analysis (PCA) and partial least-squares discriminant analysis (PLS-DA) for classifying and discriminating the treatment effects.

### 4.4. TEM Analysis

Samples were fixed overnight at 4 °C in a solution containing 2.5% glutaraldehyde and 2% paraformaldehyde in 0.1 M cacodylate buffer (pH 7.0). Following washing with 0.05 M cacodylate buffer, the samples underwent post-fixation with 1% osmium tetroxide at 4 °C for 1.5 h, followed by immersion in 0.5% uranyl acetate overnight. Subsequently, the samples were dehydrated using a series of graded ethanol solutions and embedded in Spurr’s resin, then polymerized in an oven at 70 °C for 12 h. The polymerized samples were sectioned to a thickness of 70 nm by ultramicrotome (EM UC7; Leica Microsystems, Wetzlar, Germany) and mounted onto copper 200 mesh grids. After staining with uranyl acetate for 20 min and lead citrate for 5 min, all samples were examined using JEM−1010 transmission electron microscopy operating at 80 kV (JEOL, Tokyo, Japan). Double staining with uranyl acetate and lead citrate was performed to enhance the contrast and resolution of the TEM images. The graded ethanol dehydration process was controlled to preserve sample integrity and prevent artifacts.

### 4.5. Effects of Temperature, pH, and Enzyme

To evaluate the stability of plantaricin against temperature, pH, and enzymes, a disk diffusion assay was performed using *S. aureus* ATCC 12692 as the indicator strain. The pre-cultured *S. aureus* ATCC 12692 was inoculated into a fresh medium at 1% (*v*/*v*) and incubated until the OD_600_ reached 0.5. Then, 100 μL of the culture was spread onto Tryptic soy agar (Becton, Dickinson, and Co.). A sterilized disk (paper disk for antibiotic assay, thin 8 mm, ADVANTEC, Tokyo, Japan) was placed on the agar surface, and 20 μL of synthetic plantaricin (adjusted according to temperature, pH, and enzyme treatment) was applied to achieve a final concentration of half the MIC for the total volume (0.7 μg/mL spPlnA, 0.6 μg/mL spPlnE&F, 0.8 μg/mL spPlnE&J, and 0.8 μg/mL spPlnJ&K). To examine the effect of temperature on bacteriocin activity, the bacteriocin samples were maintained in water baths at three different temperatures—20 °C, 30 °C, and 40 °C—for 12 h. To investigate the pH-dependent antibacterial activity of synthetic bacteriocins, the peptides were adjusted to pH 4, 5, 6, or 7 using a tris buffer solution (GENOMIC BASE, Namyangju-si, Republic of Korea). Proteinase–K (Protease from *Aspergillus oryzae*, P6110, Sigma-Aldrich), α–amylase (α-Amylase from *Bacillus amyloliquefaciens*, A7595, Sigma-Aldrich), and lysozyme (lysozyme chloride form from chicken egg white, L2879, Sigma-Aldrich) were prepared at a 20 mg/mL concentration to assess the stability of synthetic peptides for enzymes. The prepared enzyme was added to the synthetic plantaricin at a concentration of 4 units/g and incubated at 30 °C for 12 h. And then, to halt enzyme activity, they were heated at 100 °C for 5 min, followed by rapid cooling on ice. The antimicrobial activity was determined by measuring the size of the clear zone around the disks.

### 4.6. Statistical Analysis

The Raman peaks and antibacterial activity were analyzed using analysis of variance to determine their statistical significance (*p* < 0.05). The statistical robustness of PCA and PLS-DA was verified by Hotelling T^2^ analysis. All statistical analyses were performed using the SPSS software package version 27.0 (IBM SPSS Statistics, Armonk, NY, USA).

## 5. Conclusions

In a previous study, *L. plantarum* KM2 was isolated from low-temperature aging beef, and its complete genome sequence was analyzed [30]. Strain KM2 showed antibacterial activities against five food-borne pathogens: *Enterococcus faecalis* KCTC 2011, *S. aureus* ATCC 12692, *Escherichia coli* O157:H7 (EDL933), *Salmonella enterica* KCCM 11862, and *Vibrio parahaemolyticus* ATCC 17802. Comparative genomic analysis revealed that strain KM2 possessed the entire bacteriocin operon of plantaricins in a strain-specific manner [31]. In current studies, synthetic plantaricins based on the deduced amino acids of strain KM2 showed antibacterial activity against *S. aureus* ATCC 12692.

The above results indicate the potential of strain KM2 as an antimicrobial agent against *S. aureus*. Specifically, using strain KM2 as a starter culture in fermented foods suggests that the antimicrobial substances produced by this strain could inhibit the growth of *S. aureus*. Additionally, the culture broth of strain KM2 could be utilized as an antimicrobial agent. *L. plantarum* is known as a Generally Recognized As Safe (GRAS) microorganism, making its culture broth directly applicable to the food industry. Therefore, it can be used as an effective bio-preservative in food materials and equipment used in the food industry. Furthermore, plantaricin can be applied for even more effective use. Although all four combinations of plantaricins were effective in this experiment, applying plantaricin A alone is deemed most practical for simplicity. In conclusion, synthetic plantaricin was found to degrade the cell wall of *S. aureus*, indicating its effectiveness in inhibiting the growth of *S. aureus*. However, it is important to consider that applying plantaricin may be challenging in food processing conditions above 40 °C or in processes involving enzymes such as proteases. In the case of fermented foods, since fermentation typically occurs at lower temperatures, plantaricin is expected to be more suitable for application. However, as there is a high possibility of protein degradation, further validation experiments are necessary.

## Figures and Tables

**Table 1:** Effect of temperature, pH, and enzyme treatment on the antibacterial activity of synthetic plantaricin against *Staphylococcus aureus* ATCC 12692.

	spPlnA	spPlnE&F	spPlnE&J	spPlnJ&K
Effects of temperature				
20 °C	11.3 ± 1.2 ^a^	11.0 ± 0.0 ^a^	17.0 ± 1.7 ^b^	11.3 ± 0.6 ^a^
30 °C	11.0 ± 1.0 ^a^	11.7 ± 0.6 ^a^	13.7 ± 1.2 ^b^	9.0 ± 1.0 ^c^
40 °C	8.7 ± 0.6 ^a^	11.0 ± 0.0 ^b^	10.3 ± 0.6 ^b^	7.3 ± 0.6 ^c^
pH stability				
pH4	7.0 ± 1.7 ^a^	10.0 ± 0.0 ^b^	10.7 ± 0.6 ^b^	7.7 ± 0.6 ^a^
pH5	7.0 ± 0.0 ^a^	10.0 ± 0.0 ^b^	13.0 ± 0.0 ^c^	9.0 ± 0.0 ^b^
pH6	8.7 ± 0.6 ^a^	12.0 ± 1.0 ^b^	14.3 ± 0.6 ^c^	11.3 ± 1.5 ^b^
pH7	10.7 ± 0.6 ^a^	14.0 ± 0.0 ^a^	18.7 ± 1.2 ^a^	14.7 ± 0.6 ^b^
Effects of enzyme				
α–Amylase	11.3 ± 1.5 ^a^	12.7 ± 1.2 ^a^	11.0 ± 0.0 ^a^	12.7 ± 0.6 ^a^
Proteinase K	−	−	−	−
Lysozyme	7.3 ± 1.2 ^a^	9.3 ± 0.6 ^a^	13.0 ± 1.7 ^b^	1.7 ± 0.6 ^c^
Heat *w*/*o* enzyme	11.4 ± 0.5 ^a^	12.0 ± 0.5 ^a^	11.0 ± 0.0 ^a^	11.7 ± 1.0 ^a^

^a, b, c^ Different superscripts in a row indicate significant differences at *p* < 0.05 according to Duncan’s multiple-range test. “−” means that a clear zone was not formed.

## Data Availability

The data used to support the findings of this study can be made available by the corresponding author upon reasonable request.
